# On the Thickness Quantification of Composite Materials by Using Lock-In Thermography

**DOI:** 10.3390/ma12071185

**Published:** 2019-04-11

**Authors:** Davide Palumbo

**Affiliations:** Politecnico di Bari, Viale Japigia, 182, 70126 Bari, Italy; davide.palumbo@poliba.it

**Keywords:** thickness, lock-in thermography, non-destructive testing, composites, glass fiber reinforced plastic (GFRP)

## Abstract

Many structural components made of composite materials need an accurate thickness control during fabrication and/or maintenance. In this regard, various non-destructive techniques can be used for the online measuring of thickness of large components such as wings and fuselage in the aerospace industry. In this work, the capabilities of lock-in thermography technique in thickness measurement of glass fiber reinforced plastic material were investigated and a correct procedure has been proposed to ensure the best measurement accuracy. An analytical approach and several tests were carried out on a sample specimen with the aim to study the main test parameters. Finally, the limits of technique have been discussed.

## 1. Introduction

Composite components are used in many fields of engineering thanks to a high strength associated with the low weight. Non-destructive testing (NDT) techniques represent a useful tool for evaluating defects or damaged areas and then the residual strength of these components. 

Another important topic is represented by the verification of the geometric correctness of composite components with variable thickness. Geometrical and dimensional unconformities can occur above all during the manufacturing process and could compromise part assembling. 

NDT techniques play a key role also in thickness measurements since allow the on-line testing without destructive tests and then reducing times and costs. In this regard, different techniques can be used for thickness measurements, such as, eddy current [1], ultrasound [2], and thermography [3].

In the work of Chen et al. [1], the thicknesses of CFRP materials have been measured with eddy current method in two different way. An excitation frequency of 5 kHz has been used for measuring thickness up to 4.8 mm. The capability in measuring the thickness of the composite material as the coating of aluminum plates has been also demonstrated.

Amiri et al. [2] proposed an impact-echo method for thickness measurement of orthotropic composite plate. In particular, the response of plates subjected to low velocity impact has been used and the limits of technique has been investigated.

Lamb waves have been used in the work of Moreno et al. [4,5]. This method is based on the identification of the antisymmetrical Lamb modes in pulse propagation in plates.

All the techniques just described generally require the contact with components and a scanning approach in order to cover the whole component with consequent increasing of the testing time. In this regard, stimulated or active thermographic techniques [6] represent a valid tool for measuring the thickness of material since they provide a full-field information with a simple experimental set-up also suitable for on-line measurements. 

Pulsed (PT) [7,8,9,10,11,12] and lock-in (LT) [13,14,15,16,17,18,19,20,21,22] thermography are the techniques used in the last years for defect depth, thickness or more in general defect/damage measurement. 

The potential and limits of the flash thermography technique have been investigated by Shepard et al. [7,8]. In particular, the capability of the TSR^®^ algorithm in evaluating the thickness of Thermal Barrier Coatings has been demonstrated. Zeng et al. [9] investigated the defect depth by using a theoretical one-dimensional solution of pulsed thermography and comparing four representative methods for the non-air interface situation. 

A comparative study of pulsed and lock-in thermography was performed in the works of Shrestha et al. [21,22]. Results show that PT is faster and more accurate than LT in coating thickness measurement. 

The capability of LT in coating in defect depth and thickness measurement has been shown in several papers [14,15,16,17,18,19,20,21,22,23,24,25]. In the work of Maierhofer et al. [17] a systematic investigation has been performed on isotropic and CFRP material. In particular, it has been shown as the lateral thermal diffusion influences the results of LT technique. A new procedure for obtaining the enhancement of the depth resolution is shown in the work of Subhani et al. [18]. By using the frequency modulated thermal wave imaging, a linear relation between phase signal and depth allows for obtaining a depth resolution in defect detection of 0.2 mm. 

The limits of defect detectability were examined with LT on a steel specimen in [19]. By considering the phase contrast at a fixed excitation frequency, the defect detectability shows an exponential trend as function of the defect depth. In the work [20] has been shown as a third order polynomial regression can be used to relate the coating thickness and the phase signal. The proposed procedure allows to obtain an error of about 5% in thermal barrier coating thickness measurement. Substantially, two methods have been used for defect depth or coating thickness prediction: the blind frequency method and the phase contrast method, each of one with own limits and peculiarities. In fact, this topic has been less explored than PT technique in literature. Moreover, the papers in which the accuracy has been evaluated, do not describe the procedure or method to choose the best excitation frequency (or period). 

In this work, the capabilities of LT technique in thickness verification of composite material were investigated and a procedure is proposed to choose the best excitation frequency (period) in order to estimate the resolution in thickness measurement variation. An approach based on analytical models and experimental tests have been carried out by considering a sample specimen made of glass fiber reinforced plastic (GFRP) material. Finally, the limits of technique have been discussed.

## 2. Theory

Lock-in thermography is based on the generation of thermal waves inside the specimen, for example, by periodically exciting the specimen surface [6]. The resulting oscillating temperature field in the stationary regime can be recorded remotely through its thermal infrared emission by an IR camera. Thermal wave can be reconstructed by measuring temperature evolution over the specimen surface: by a suited algorithm, information about magnitude and phase of the thermal wave can be obtained.

In the works of Rosencwaig et al. [23] and Bennet et al. [24], the theory of the photoacoustic effect and then of thermal wave interferometry has been presented by means a one-dimensional model of the heat flaw in the cell resulting from the adsorbed light energy. If a sinusoidal heat source is considered, the amplitude modulus (*A*) and the phase (φ) difference between the reference and the sample signals (Phase) are given by [24]:

(1) $$A=\sqrt{\frac{{\left[\frac{1+R_{2}e^{-2h_{n}}}{1-R_{1}R_{2}e^{-2h_{n}}}\right]}^{2}-\frac{F\sin^{2}\left(h_{n}\right)}{R_{1}}}{1+F\sin^{2}\left(h_{n}\right)}}\;\varphi =arctg\left\{\frac{-R_{2}\left(1+R_{1}\right)e^{-2h_{n}}\sin\left(2h_{n}\right)}{1-R_{1}{\left(R_{2}e^{-2h_{n}}\right)}^{2}+R_{2}\left(1-R_{1}\right)\cos\left(2h_{n}\right)}\right\}$$

where

(2) $$F=\frac{4R_{1}R_{2}e^{-2h_{n}}}{{\left[1-R_{1}R_{2}e^{-2h_{n}}\right]}^{2}},\;h_{n}=\frac{h}{\mu },\;\mu =\sqrt{\frac{2\alpha }{\omega }}=\sqrt{\frac{\alpha }{\pi f}}=\sqrt{\frac{\alpha }{\pi }T}$$

with *h* specimen thickness, *μ* thermal diffusion length, *h_n_* normalized thickness, *R*_1_ and *R*_2_ reflection coefficients at the surface and at the interface to the defect, respectively [24]. *α* is the thermal diffusivity, *f* and *T*, the modulation frequency and period. 

It is important to underline as Equation (1) are valid if the specimen is thermally thick [23] and in the case of 1-D heat transfer model. 

## 3. Material and Methods

### 3.1. Material and Experimental Set-Up

**Figure 1:**
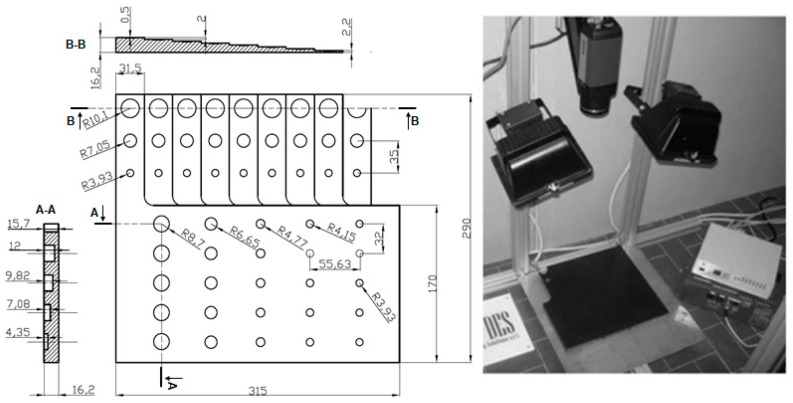
Glass fiber reinforced plastic (GFRP) specimen and experimental set-up.

A specimen consisting in a plate with simulated defects was manufactured by means of the vacuum infusion resin process. An epoxy-type (i.e., EC 157 by ELANTAS) resin was employed, reinforced with a double layer of quadriaxial glass fiber of the type 0°/+45°/90°/−45°, Figure 1. In Table 1 are reported the thermo-physical constants of material (GFRP). 

Thermography tests were performed by IR camera FLIR A655sc (FLIR^®^ Systems, Inc., Wilsonville, OR, USA) with thermal sensitivity (NETD) < 30 mK and based on a micro-bolometer detector with 640 × 480 pixels.

The set-up used is shown in Figure 1 (reflection mode). Two halogen lamps with a power of 500 W were controlled by MultiDES^®^ system (Des srl, Bari, Italy) in order to heat specimens with a series of square waves. IR camera and lamps were placed to 980 mm and 560 mm from the specimen, respectively. The geometrical resolution obtained is 0.65 mm/pixel.

Thermal data were processed by IRTA^®^ software [12,13,26] in order to obtain amplitude and phase images from lock-in tests. IRTA algorithms [26] for lock-in take into account the thermal transient in the very first cycles. So, there is no need to reach stationary behavior of the thermal response of the material to the excitation source.

A modulated square wave has been used as a heat source in order to reduce the testing time. In fact, as demonstrated in [12], the thermal response of material contains information about higher frequencies proportional to the main frequency. Several tests have been carried out as shown in Table 2. Three exciting cycles were acquired for each sequence with different frame rate depending on the modulated period, as indicated in Table 3.

By decomposing the thermal response in time domain as the sum of a singular sinusoidal wave, we can write:

(3) $$T_{m}(t)=a+bt+\sum_{n=1}^{\infty }\Delta T_{n}\sin(n\omega t+\varphi_{n})\;n=1,3,5,7$$

where *t* is the time, *a* and *b* are two constants used to model the average temperature growth of the material, *ω* is the modulated frequency of the main harmonic (first Fourier component), ∆*T_n_* and $\varphi_{n}$ are the amplitude and the phase angle (as defined in Equation (1)) of *n*-th Fourier component. As it shown in Equation (3), any odd number (*n*) can be set to obtain information about the relative Fourier component. However, the Fourier components will be gradually affected by a higher noise level as *n* value increases. So, in this paper the analysis has interested only the first, third and fifth Fourier component. IRTA software allows for obtaining information about the first, 3-rd and 5-th Fourier components from the analysis of one thermal sequence by setting the values of harmonics [12]. 

**Figure 2:**
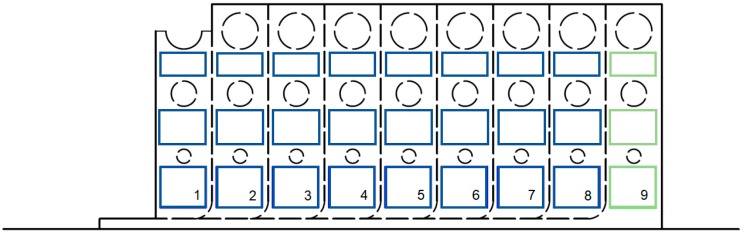
Areas (ROIs) used on each step for phase signal evaluation.

The phase data derived from the analysis with the IRTA software were then processed with MATLAB software (R20171b, The MathWorks, Inc., Natick, MA, USA) in order to extract for each step the phase information. In particular, one area (ROI, Region Of Interest) has been considered for each step, as shown in Figure 2 (areas with the flat bottom holes have been excluded) and the mean and standard deviation values have been calculated. The last step (green ROIs in Figure 2) has been used as reference one. So, the phase values for experimental data for each step are:

Phase = mean(Phase)_ROIi_ − mean(Phase)_ROI9_ (4)

where ROIi refers to the ROI of i-th step.

In Table 4, is reported the thickness of each step (the blind holes are not considered).

### 3.2. Proposed Procedure

In this section, a new method/procedure is proposed for identifying the best frequency/period value that allows to verify the thickness with the best accuracy. The proposed procedure starts from the model proposed by Rosencwaig [23] and Bennet [24].

**Figure 3:**
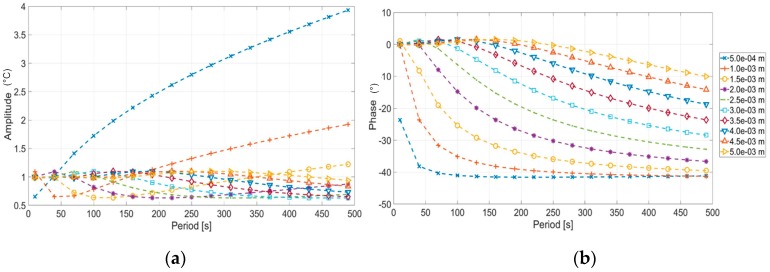

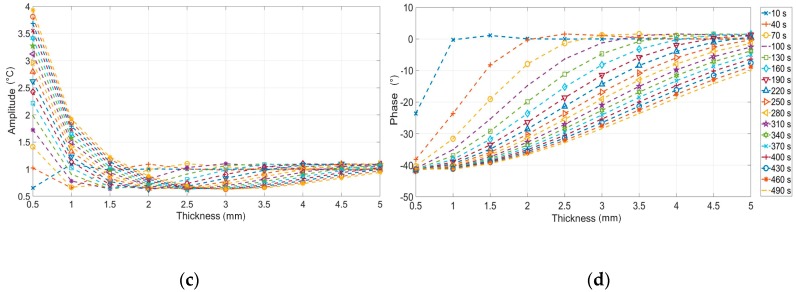
Amplitude and phase signal derived with Equation (1): (**a**) Amplitude signal versus period, (**b**) phase signal versus period, (**c**) amplitude signal versus thickness, and (**d**) phase signal versus thickness.

In Figure 3, the theoretical trends of Amplitude (*A*) and Phase (*φ*) obtained from Equations (1) and (2) are shown. The explored thicknesses and modulated periods used for the analytical simulation by using Equations (1) and (2). 

Figure 3c, shows as the amplitude signal decreases very quickly as the thickness increases. This behavior makes the amplitude signal less suitable for evaluating deeper defects. In this regard, the phase signal is considered more appropriate for investigating the defects depth or thicknesses with the lock-in technique [15,17,25].

As shown in Figure 3, each thickness presents a characteristic value of the period in which the phase signal become zero. This occurs in correspondence of the so called “blind frequency” [15,16,17] (in our case, blind period). Moreover, for a fixed value of the period, each thickness presents different values of phase signal.

Blind frequency and phase contrast methods are widely used in literature [15,16,17] for depth or thickness quantification by means of lock-in thermography. The first method is based on the following equation [15]:

(5) $$h=C\sqrt{\frac{\alpha }{\pi f_{b}}}=C\sqrt{\frac{\alpha }{\pi }T_{b}}$$

where *C* is a constant that depends on material and can be derived from experimental tests and *f_b_* and *T_b_* are the blind frequency and period respectively. This method is not immediately applicable since it is not easy to find the blind frequency from the thermographic data. Moreover, it has been demonstrated as the blind frequency depends on the defect size, shape and depth [15]. 

The phase contrast method is based on the phase contrast values evaluation in correspondence of a single value of frequency/period. Also, this method requires an accurate calibration procedure for obtaining acceptable errors in depth evaluation [15,20].

A different point of view is provided in this work since the attention was focused on verifying the thickness or more in general the geometry of components made of composite material. So, the focus is the excitation frequency (period) and the method or procedure to obtain it to estimate the resolution in thickness measurement variation.

By starting to the phase signal trend as function of the thickness, Equation (1) and Figure 3, it is simple to notice as for a fixed value of period, the phase presents a point in which its variation with respect to thickness is maximum. This point coincides with the one in which the first derivative of phase is maximum. In other words, this means that for a fixed thickness value, the best period is the one that provides the maximum of the first derivative of the phase signal. In fact, in this condition, the higher sensitivity is obtained since phase signal variations will be measured also in correspondence of little thickness variations. 

Starting from Equation (1), the first derivative of the phase signal is:

(6) $$\frac{d\varphi }{dh_{n}}=-\frac{f_{1}\left(h_{n}\right)-f_{2}\left(h_{n}\right)+f_{3}\left(h_{n}\right)}{f_{4}\left(h_{n}\right)}$$

with

(7) $$f_{1}\left(h_{n}\right)=\frac{2R_{2}\left(1+R_{1}\right)\cos\left(2h_{n}\right)e^{-2h_{n}}}{R_{2}\left(1-R_{1}\right)\cos\left(2h_{n}\right)-R_{1}e^{-4h_{n}}{\left(R_{2}\right)}^{2}+1}$$

(8) $$f_{2}\left(h_{n}\right)=\frac{2R_{2}\left(1+R_{1}\right)\sin\left(2h_{n}\right)e^{-2h_{n}}}{R_{2}\left(1-R_{1}\right)\cos\left(2h_{n}\right)-R_{1}e^{-4h_{n}}{\left(R_{2}\right)}^{2}+1}$$

(9) $$f_{3}\left(h_{n}\right)=\frac{R_{2}\left(1+R_{1}\right)\sin\left(2h_{n}\right)e^{-2h_{n}}\left[2R_{2}\left(1-R_{1}\right)\sin\left(2h_{n}\right)-4R_{1}{\left(R_{2}\right)}^{2}e^{-4h_{n}}\right]}{{\left[R_{2}\left(1-R_{1}\right)\cos\left(2h_{n}\right)-R_{1}e^{-4h_{n}}{\left(R_{2}\right)}^{2}+1\right]}^{2}}$$

(10) $$f_{4}\left(h_{n}\right)=\frac{{\left[R_{2}\left(1+R_{1}\right)\right]}^{2}\sin{\left(2h_{n}\right)}^{2}e^{-4h_{n}}}{{\left[R_{2}\left(1-R_{1}\right)\cos\left(2h_{n}\right)-R_{1}e^{-4h_{n}}{\left(R_{2}\right)}^{2}+1\right]}^{2}}+1$$

**Figure 4:**
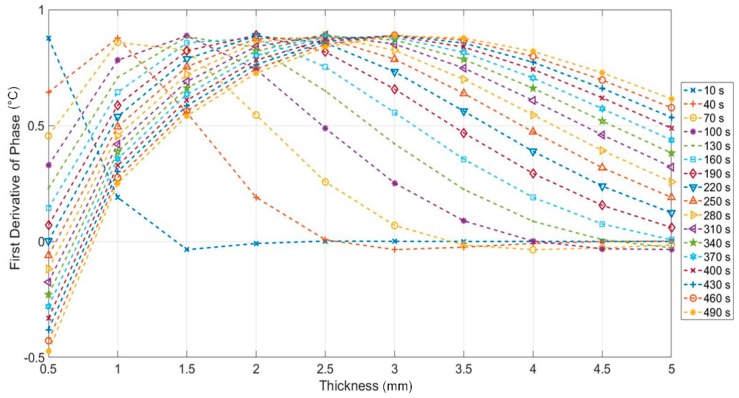
First derivative of the phase signal versus thickness for different value of periods (Equation (4)).

In Figure 4 is shown the first derivative phase signal as function of thicknesses and for different values of period, Equation (4). This figure allows for obtaining for each investigating thickness, the value of period in which the sensitivity of the phase signal is maximum. As expected, the best period increases if the thickness increases. 

It is interesting to notice as, for a fixed value of thickness, the first derivative of the phase (Figure 4) and the phase (Figure 3d) reach the maximum value in correspondence of different values of period. As example, by comparing Figure 3 and Figure 4, in correspondence of 1.5 mm, the maximum of the first derivative signal is reached for 100 s but, a better phase signal is obtained with higher periods. In other words, for a fixed value of the thickness, the best period allows to obtain the best phase sensitivity and not the higher phase signal.

## 4. Results and Discussion

In this section, results of the experimental tests and a comparison with the simulation are shown in order to verify the proposed approach.

**Figure 5:**
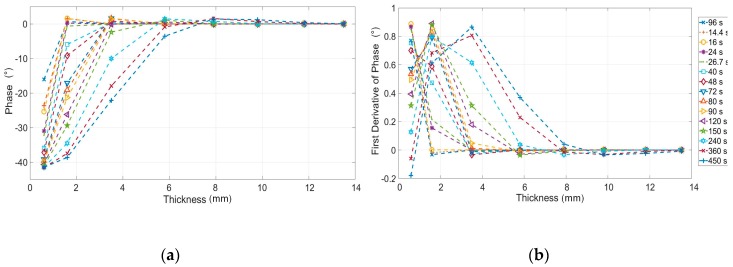
(**a**) Phase signal and (**b**) first derivative of the phase signal versus signal for the thicknesses investigated with simulation (Equations (1) and (4)).

Simulations were carried out by adopting Equations (6)–(10), the material constants in Table 1, modulation periods shown in Table 2 and thicknesses in Table 3. In Figure 5 are reported the results in terms of phase and first derivative signal. The last step (thicker) has not been reported because considered as reference to obtain the phase signal from experimental data. As expected, the last period (450 s) allows to investigate depth until about 6 mm. 

It is worth to notice as, in this case, the first derivative of the phase describes for each thickness the capability to discern contiguous thickness variations. 

**Figure 6:**
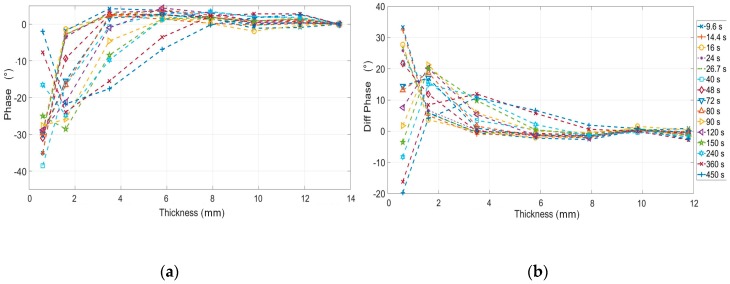
(**a**) Phase signal and (**b**) differencing of the phase signal (approximate derivative) versus signal for the thicknesses investigated with the sample specimen (experimental results).

**Figure 7:**
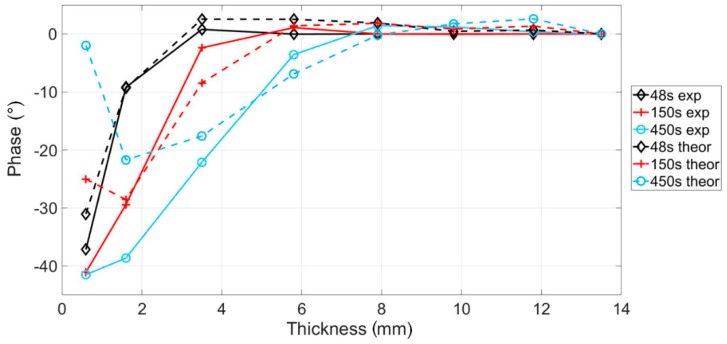
Comparison between theoretical (continuous lines) and experimental results (dotted lines) for three values of modulation period (48 s, 150 s, 450 s).

Very similar trends were obtained from the experimental data in terms of phase signal (Figure 3a and Figure 5a). However, it is clear as (Figure 6a) different values of the phase were obtained in correspondence of higher periods (from 150 to 450 s). In Figure 7 is shown a comparison between theoretical and experimental data for three values of the modulation period. As expected, the phase signal values obtained from experimental data are lower than simulations ones. In particular, results differ as the modulation period increases. Indeed, in these conditions the hypotheses of the 1-D model used for simulations are no longer valid since, the in-plane heat diffusion cannot be neglected, and the specimen cannot be considered thermally thick.

To obtain a comparison with simulations in terms of first derivative signal, an approximate derivative of experimental data has been calculated (named Diff Phase) by simply differencing adjacent values of phase. Figure 6b provides immediately an indication of the best periods and of investigable thicknesses. In this regard, it is important to underline as the analysis of the signal to noise ratio is necessary to know the sensitivity of the phase signal for the thickness variations measurement.

Various sources of noise can affect the phase signal although it is less susceptible than amplitude to non-uniform heating and surface irregularities. They depend on the experimental set-up and the state of the specimen surface. In the first case, a non-uniform heating of the surface due to an incorrect lamp positioning or incorrect lamps operation can induce phase signal variations. In particular, a non-uniform heating of the specimen can generate significant in-plane heat diffusion with consequent in-plane phase variations. 

In the second case, the surface can present local emissivity variations due to scratches or little humps and cavities. These irregularities also represent a local variation of the absorption coefficient and then local non-uniform heating. In any case, it is not simple to predict the noise of the phase signal. The noise should be evaluated from time to time depending on the particular application. Moreover, it is worth underlining as an additional source of noise can be added to the algorithm used for the signal processing to obtain phase data. In other words, each algorithm presents an own characteristic level of noise. However, a simple way for estimating the noise is to consider the Standard Deviation of the phase signal. 

**Figure 8:**
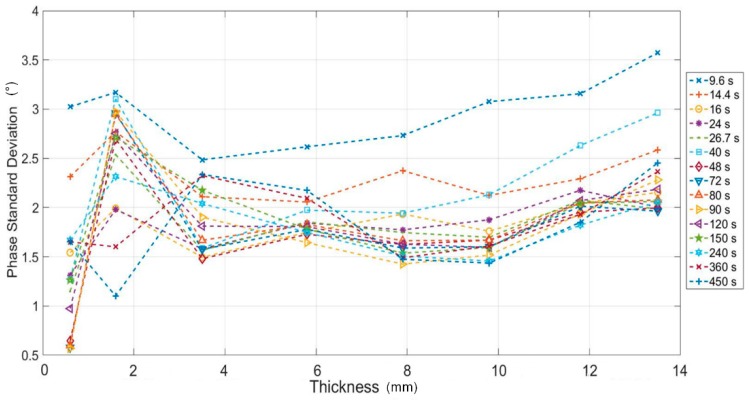
Phase Standard Deviation versus thickness of each step for different values of the modulation period.

In Figure 8, the value of the phase Standard Deviation (SD) is reported for each area (ROI) of each step and for different values of the modulated period. As expected, the SD decreases while period increase since long periods means more heat and then more signal with respect to the noise. The SD increases as the thickness increases. Also, in this case, for a fixed period, higher thicknesses give a lower value of the signal to noise ratio.

If we consider the mean value as representative of the phase SD, 2.5° can be considered as conservative value. In this regard, a threshold value equal to three times the phase SD can be considered for evaluating the minimum phase signal associated with the thickness variation. In other words, a phase signal of about 8° is necessary for measuring a thickness variation.

**Figure 9:**
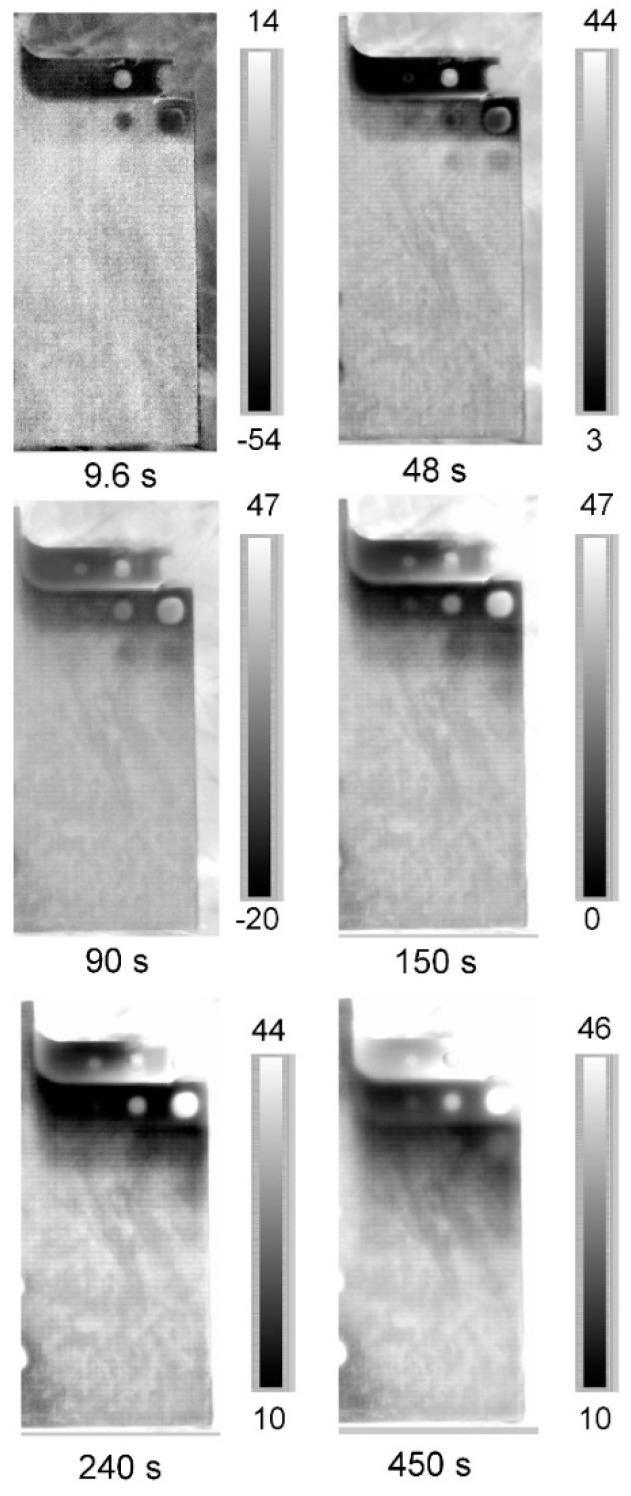
Phase maps of the steps for different values of the modulation period.

In this way, it is immediate from Figure 6 to understand which thickness variations can be measured by the phase data (thickness resolution). As expected, the sensitivity of lock-in technique and then of the phase signal, decreases as the thickness increases. It follows that with adopted periods the range of measurable thickness is 0.6–5.8 mm. It is important to underline as these results depend on the adopted set-up and test parameters. These results are confirmed in qualitative way form the Figure 9 in which the phase maps at different modulated period are reported. Theoretically, higher periods would allow to see more deeper thickness (as predicted from Equation (1)) but, as already said, the in-plane diffusivity limits the investigable thicknesses.

**Figure 10:**
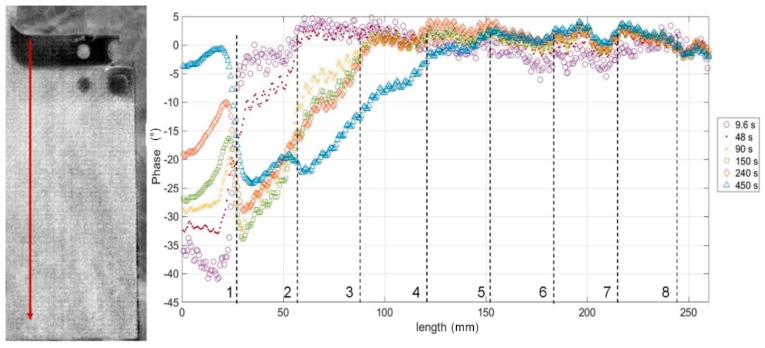
Phase signal (φ) along the steps for different values of the modulation period.

In Figure 10 the phase profile along steps is reported for 6 values of periods. It has been obtained as mean value of 20 phase profiles along the red line in Figure 10. It is clear the effect of the in-plane diffusivity that does not allow to reach a steady-state value of phase within each step and reduces the sensitivity with respect to the theoretical model. 

Summarizing, the first derivative of the phase signal can represent a useful tool for detecting the best period for verifying the thickness. In this regard, a procedure can be set-up to obtain information the measurable thickness variations with phase data. The nominal values of thickness are known.

- Firstly, if the material constants are known, the 1-D model can be used for estimating the correct periods in terms of the phase and first derivative of the phase. This latter provides information about the period for which the phase signal has the maximum sensitivity. To obtain a better accuracy in evaluation of the best period, diffusivity and effusivity of material can be retrieved from a sample specimen by means of experimental tests.
- Evaluating the noise of the technique by considering the Standard Deviation of the phase signal on a sample specimen.
- Estimating the sensitivity of technique by fixing a threshold value equal to two or three time the value of SD.
- Estimating the thickness variations that can be measured by the phase data.
- Verifying the thickness or thickness variations by means of the phase signal by adopting the best period.

It is important to underline as if a sample specimen is not available, the described procedure is still valid, but the accuracy will be strongly related to the hypothesis of 1-D model (semi-infinite plate and thermally thick specimen). In this case, the noise must to be estimated by measuring the SD on the same component under analysis.

## 5. Conclusions

In this work the capabilities of lock-in thermography technique in thickness verification of composite plates were investigated. In particular, a procedure has been proposed for estimating the best periods of excitation, based on the first derivative of the phase signal.

A sample specimen made of GFRP material has been used to verify the results obtained by using a theoretical 1-D model. The main results obtained were:

- the possibility to estimate the resolution in thickness measurement variation as a function of the thickness by using a 1-D model,
- the accuracy (in period evaluation) of the proposed method depends on the hypothesis of the 1-D theoretical model,
- proposed approach can be a useful tool in the case of a sample specimen is not available. The first derivative analysis and then the best period can be obtained by estimating the thermo-physical constants on the same component under analysis since the nominal thicknesses are known.

## Figures and Tables

**Table 1:** Thermo-physical constants of material, explored thicknesses, and modulated periods used for simulation.

*k* (W/m K)	*ρ* (kg/m^3^)	*cp* (J/kg K)	*α* (m^2^/s) × 10^−7^	*R*_1_ = *R*_2_	*h* (m) × 10^−3^	*T* (s)
0.3	1900	1200	1.3	0.99	0.5–5 (step 0.5)	10–490 (step 10)

**Table 2:** Parameters used for lock-in thermography (LT) tests.

Test Number	Modulation Period (s)	Modulation Frequency (Hz)	Heat Source	Number of Cycles	Harmonics	
1	48	0.021	square	3	0.021; 0.062; 0.105 (Hz)	48; 16; 9.6 (s)
2	72	0.014	square	3	0.014; 0.042; 0.069 (Hz)	72; 24; 14.4 (s)
3	80	0.013	square	3	0.013; 0.038; 0.063 (Hz)	80; 26.7; 16 (s)
4	120	0.008	square	3	0.008; 0.025; 0.042 (Hz)	120; 40; 24 (s)
5	240	0.004	square	3	0.004; 0.013; 0.021 (Hz)	240; 80; 48 (s)
6	360	0.003	square	3	0.003; 0.008; 0.014 (Hz)	360; 120; 72 (s)
7	450	0.002	square	3	0.002; 0.007; 0.011 (Hz)	450; 150; 90 (s)

**Table 3:** Acquisition parameters used for lock-in tests.

Test Number	1	2	3	4	5	6	7
Frame rate (frame/s)	3	2	2	1	1	1	1
Total frames (3 cycles)	432	432	480	720	720	1080	1350

**Table 4:** Thickness of each analyzed step of the sample specimen.

Step	1	2	3	4	5	6	7	8	9
Thickness (mm)	0.6	1.6	3.5	5.8	7.9	9.8	11.80	13.50	16.2
